# MET Oncogene Targeting for Cancer Immunotherapy

**DOI:** 10.3390/ijms25116109

**Published:** 2024-06-01

**Authors:** Andrea Maria Lombardi, Dario Sangiolo, Elisa Vigna

**Affiliations:** Department of Oncology, University of Torino, 10043 Torino, Italy; andreamaria.lombardi@unito.it (A.M.L.); dario.sangiolo@unito.it (D.S.)

**Keywords:** MET oncogene, MET antibody, MET tyrosine kinase inhibitors, targeted therapy, immunotherapy, cellular immunotherapy

## Abstract

The MET receptor is one of the main drivers of ‘invasive growth’, a multifaceted biological response essential during embryonic development and tissue repair that is usurped by cancer cells to induce and sustain the malignant phenotype. MET stands out as one of the most important oncogenes activated in cancer and its inhibition has been explored since the initial era of cancer-targeted therapy. Different approaches have been developed to hamper MET signaling and/or reduce MET (over)expression as a hallmark of transformation. Considering the great interest gained by cancer immunotherapy, this review evaluates the opportunity of targeting MET within therapeutic approaches based on the exploitation of immune functions, either in those cases where MET impairment is crucial to induce an effective response (i.e., when MET is the driver of the malignancy), or when blocking MET represents a way for potentiating the treatment (i.e., when MET is an adjuvant of tumor fitness).

## 1. Introduction

**Figure 1:**
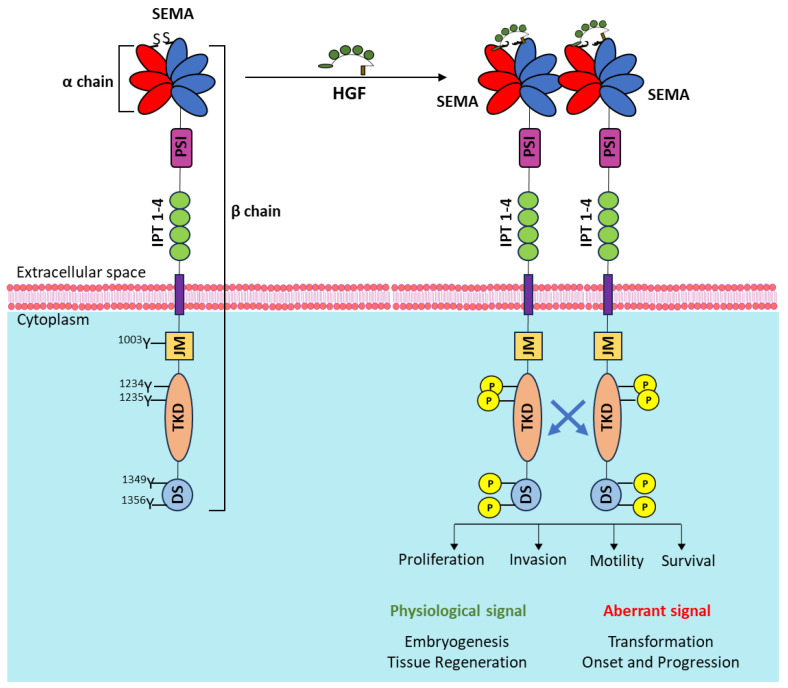
Schematic representation of the MET receptor (**left**) and its activation upon ligand interaction, eliciting MET phosphorylation and triggering intracellular signaling responses (**right**). SEMA: semaphorin domain; blades 1–3 (in red) constitute the α-chain; blades 4–7 (in blue) belong to the β-chain; PSI: plexin–semaphorin–integrin homology domain; IPT 1–4: four immunoglobulin–plexin–transcription factor domains; JM: juxtamembrane domain; TKD: tyrosine kinase domain; DS: docking site. Relevant tyrosine residues (in yellow if phosphorylated) are highlighted.

The *MET* gene, located on chromosome 7q21–q31 [1], encodes for a transmembrane tyrosine kinase, the receptor for hepatocyte growth factor/scatter factor (HGF/SF) [2]. Synthetized as a single-chain precursor, the mature protein form is a heterodimer with an extracellular α subunit linked via disulfide bonds to a β transmembrane subunit [3]. The extracellular domain comprises a semaphorin (SEMA) domain, a plexin–semaphorin–integrin (PSI) homology domain, and four immunoglobulin–plexin–transcription factor (IPT) domains. The intracellular part of MET is composed of a short juxtamembrane domain including the tyrosine at position 1003 responsible for the interaction with c-CBL [4], followed by the tyrosine kinase (TK) domain, and a tail region. The TK domain contains two tyrosine residues at positions 1234 and 1235, representing the major phosphorylation sites [5], while two additional tyrosines (positions 1349 and 1356) located in the tail represent the docking site for signal transducers [6]. Upon interaction with its unique high-affinity ligand, HGF, MET kinase activity turns on, resulting in MET receptor transphosphorylation and activation of multiple intracellular signaling pathways. As MET is expressed by cells of epithelial origin, while HGF is expressed by cells of mesenchymal origin, the stimulation of the receptor by the ligand occurs via a paracrine mechanism [7,8]. MET signaling elicits multifaceted biological responses such as cell motility, growth, invasion, and regulation of apoptosis [9]. These functions regulate physiological processes such as embryogenesis, organ development, tissue regeneration, homeostasis, and wound healing [10,11,12,13]. When MET signaling is aberrant and deregulated, it promotes proliferation, invasiveness, and survival, sustaining both cancer onset and progression [14,15] and being crucial during the formation of distant metastasis [16] (Figure 1).

MET dysregulation occurs in the presence of *MET* gene genetic or transcriptional alterations [17,18]. The first group includes *MET* gene amplification, mutations, and fusions. *MET* gene amplification, occurring at a rate of around 4% across various tumor types, arises from polysomy or focal copy number gain. *MET* gene amplification generates overexpression and ligand-independent MET receptor activation [19]. High levels of MET amplification give rise to oncogene addiction [20,21]. Preclinical studies in vitro and in vivo suggest that a threshold of five copies of the *MET* gene is required for addiction. However, from the clinical point of view, such a cut-off is hardly established, making it difficult to stratify patients responsive to MET-targeted therapies effectively. Of note, MET amplification often represents a mechanism to sustain secondary resistances to epidermal growth factor receptor (EGFR) targeting agents in Non-Small-Cell Lung Cancer (NSCLC) and colorectal cancer (CRC) [22,23]. Activating point mutations have been found in different types of cancer patients. They are located in the SEMA domain, interfering with ligand interaction/activation, in the juxtamembrane domain, concurring in disrupting negative receptor regulations, and in the kinase domain, activating the catalytic function and inducing resistance to anti-MET therapy by Tyrosine Kinase Inhibitors (TKIs) [24]. Peculiar mutations occur in the non-coding region of the *MET* gene, involving acceptor–donor splicing sites and generating an alternatively spliced receptor lacking the entire juxtamembrane domain (MET exon 14 skipping mutants) [25]. MET exon 14 skipping is more frequently found in NSCLC patients and is considered an oncogenic driver [26]. Indeed, MET TKIs are approved for treating these patients [27,28]. Nevertheless, only some of them benefit from the therapy [29,30], suggesting that alternative treatments are required. Fusions are rare events occurring intra- or inter-chromosome 7. These chimeric hybrids, generating either transmembrane or intracellular molecules, dimerize in a ligand-independent fashion, sustaining constitutive kinase activation [31].

Transcriptional alterations can involve the receptor or the ligand. MET overexpression is a frequent event in cancer [32,33] and is associated with poor prognosis [34,35,36,37,38,39]. It occurs as a mechanism to overcome stress events and bypass barriers during cancer progression [40]. MET overexpression gives rise to receptor activation that can be further overstimulated by the ligand [41,42]. HGF overexpression has been associated with resistance to EGFR-targeted therapies [43]. Finally, autocrine loops characterized by the co-expression of MET and HGF by the same cell are frequently found in non-epithelial tumors [44,45,46] and in gliomas [47,48].

## 2. Overview of MET-Targeting Molecules

Due to the pivotal role of MET in cancer, the use of agents targeting MET is strongly envisaged. As of now, small molecule TKIs, recombinant inhibitory antibodies endowed with different mechanisms of action, antibody–drug conjugates (ADCs), and synthetic molecules such as Chimeric Antigen Receptors (CARs) suitable to elicit an immune response against MET-expressing tumors have been developed. Considering the small molecules inhibiting MET kinase activity, specific or multi-targeting drugs have been developed and tested in the clinic. The first group includes Capmatinib, Tepotinib, and Savolitinb, which are approved for the treatment of NSCLC patients featuring MET exon 14 skipping [27,28]. The second group, i.e., the multi-targeting drugs, comprises Crizotinib, authorized for the treatment of ALK and Ros-1-positive NSCLC [49], and Cabozantinib applied for advanced thyroid tumors [50]. These two molecules have also been explored for blocking MET activation in clinical trials or case reports including cancer patients featuring MET activation [51,52,53,54,55,56,57,58,59,60,61,62,63,64,65]. With regards to MET antibodies suitable to inhibit MET signaling, they act through different mechanisms: (i) by blocking the interaction between MET and HGF; (ii) by inhibiting MET dimerization; (iii) by accelerating MET turnover; iv) by downregulating MET from the cell surface; and (v) by enzymatically removing MET from the cell surface. Some of them displayed more than one of the above-listed activities concomitantly (see Table 1). Notably, antibodies that exclusively block the interaction between the ligand and the receptor at the high-affinity binding site, such as the anti-HGF Rilotumumab [66] and the monovalent anti-MET Onartuzumab [67], have been tested in the clinic without great success [68,69,70] and their application has been dismissed. The reason for this failure is multifactorial, but one major point is related to the fact that ligand-dependent MET activation does not sustain MET addition. In fact, a necessary condition to elicit a therapeutic response from agents acting exclusively by impairing MET-mediated intracellular signaling is that *MET* must be the driver gene of the malignancy. Other molecules, such as MET-ADCs and MET-CARs, can be effective independently of the mechanisms that sustain the malignancy. The major requirement for their efficacy is a good level of surface MET expression. As mentioned above, MET overexpression is quite common in carcinomas, and thus these molecules potentially have a large spectrum of applicability. Telisotuzumab Vedotin (ABBV-399) [71] and REGN5093-M114 [72] are currently under evaluation in NSCLC patients (NCT05513703; NCT04928846; NCT02099058; NCT06093503; NCT04982224; see Supplementary Table S1 for additional information about these trials). The application of ADCs requires a careful evaluation of the dose needed to reach a good therapeutic response without eliciting adverse effects due to the conjugated payload. MET-CARs, as well as MET-targeting molecules exhibiting inhibitory properties linked to an immune response modulation, either as single agents or in combination, are discussed in detail below.

## 3. MET-Targeting Antibodies to Induce Immune-Mediated Cancer Cell Death

Antibodies are molecules intrinsically able to concur with the endogenous immune system in fighting cancer cells. Some of the MET-inhibitory antibodies display immune-mediated cancer cytotoxicity. These functions, either natural or enhanced by genetic modifications, are relevant to the final therapeutic outcome of the molecule. Examples of such antibodies are described below.

Sym015 is a mixture of two humanized IgG1 antibodies recognizing non-overlapping epitopes located in the MET SEMA domain [73]. Sym015 blocks ligand/receptor interaction by competing with the β chain of HGF for SEMA blade2/blade 3 binding. The correct interaction between the HGF β chain and MET is required to elicit HGF-driven biological responses [74]. In addition, Sym015 treatment promotes MET internalization followed by lysosomal-dependent degradation, significantly reducing the amount of MET receptor exposed at the cell surface. These two distinct functions allow potential inhibition of intracellular signaling in both ligand-dependent and -independent MET activation [73]. Thanks to the IgG1 backbone of the antibodies included in the mixture, Sym015 elicits Antibody-Dependent Cellular Cytotoxicity (ADCC) and Complement-Dependent Cytotoxicity (CDC). These functions crucially contribute to the Sym015 anti-tumor activity, as proved by including in the IgG1 backbone two single point mutations suitable to abolish ADCC and CDC effector functions (i.e., Sym015-LALA) [73]. After efficacy validation in preclinical models characterized by MET amplification, overexpression, or exon 14 deletion and assessment of preclinical pharmacology/safety in non-human primates [75], Sym015 has been evaluated in a Phase 1a/2a trial to investigate the safety, tolerability, and antitumor activity in patients with advanced solid tumor malignancies (NCT02648724). The trial is now closed and the results are under evaluation.

ARGX-111 is an engineered antibody derived from the WT52 antibody. WT52, interacting with MET SEMA blade2/blade3, has been selected from a panel of chimeric llama–human antibodies based on the most powerful inhibitory activity [76]. WT52 competed with HGF for MET binding and induced MET internalization. The llama sequences of the WT52 framework variable regions were compared to the human framework germline and sequences with 95% identity were selected and introduced in the IgG1 human backbone. This was further modified by introducing point mutations: (i) in the CH2 region, to enhance ADCC function; (ii) in the CH3 region, to enhance antibody recycling from the sorting endosome (i.e., NHance^®^ mutations). In addition, ARGX-111 was produced in an afucosylated form to increase affinity for FcγRIIIa, thus further potentiating ADCC. The therapeutic function of ARGX-111 has been validated in HGF-dependent and -independent tumor models in vivo. These studies assessed that the specific engagement of natural killer (NK) cells against MET-expressing cancer cells provides advantages over simple MET signaling inhibition [77]. ARGX-111 was evaluated in a phase 1b trial to determine dose-limiting toxicity in patients with advanced cancers overexpressing MET (NCT02055066). The antibody showed an overall favorable safety profile up to 3 mg/kg, and stabilization of the disease in 46% of the treated patients [78].

Amivantamab (JNJ-61186372) is a fully human IgG1 bispecific antibody (BsAb) targeting EGFR and MET. The molecule has been conceived based on the well-documented cross-talk between these two receptors and the compensation of either individual receptor signaling when the other one is inhibited. Indeed, EGFR and MET signaling pathways sustain reciprocal resistance upon respective TKI treatment. Amivantamab was selected from a large panel of BsAbs as the optimal molecule with the potential to concomitantly inhibit EGFR and MET pathways. The antibody MET arm binds to the MET SEMA domain, competing with the HGF β chain while the EGFR arm interaction has been mapped to EGFR domain III, interfering with EGF binding [79]. Of note, the MET arm binds to the target with high affinity (Kd = 40 pmol/L), while the EGFR arm interacts with low affinity (Kd = 1.4 nmol/L). This supports a preferential interaction of Amivantamab with MET that, as a second step, binds also to EGFR. The two-step interaction renders the binding to cells expressing physiological EGFR levels unlikely. This reduces the possibility of Amivantamab being engaged by not-transformed cells, potentially lowering toxic effects. Similarly to ARGX-111, Amivantamab production is established in cells incorporating low levels of fucose in the Fc region of the molecule, potentiating natural killer-mediated ADCC. In addition to ADCC, phagocytosis (ADCP) and trogocytosis (ADCT) are also activated as a consequence of the antibody Fc domain interaction with the Fc receptor expressed by monocytes and macrophages. ADCT, consisting of the tumor-targeted, antibody-mediated transfer of membrane fragments from tumor cells to effector cells, significantly contributes to the removal of both EGFR and MET from the cancer cell surface. This mechanism—first described for a therapeutic antibody—represents the dominant way by which Amivantamab induces tumor remission [80]. Preclinical models in vitro and in vivo highlight that the Fc-mediated effector functions of Amivantamab are essential for its maximal antitumor efficacy. The simultaneous binding of Amivantamb to EGFR and MET drove the design of clinical trials in which the patients should benefit from the concomitant inhibition of the signaling triggered by both receptors. Nevertheless, in principle, Amivantamab should also be applied for blocking only one of the two, in particular the MET signaling. Considering the higher affinity of the antibody for MET binding, and that, thanks to the Amivantamb bispecific structure, only one antibody arm interacts with MET, potential MET-agonistic properties of the antibody are avoided. This must be considered an advantage because HGF-mimicking activity represents a recurrent problem encountered during the development of MET-inhibitory antibodies. Amivantamab has been tested in the clinic in different phase III clinical trials including NSCLC patients. The PAPILLON trial (NCT04538664) highlighted the superior efficacy of Amivantamab in combination with chemotherapy as compared to chemotherapy alone for the first treatment of non-classical EGFR exon 20-mutated disease [81]. The MARIPOSA trial (NCT04487080) was designed to compare Osimertinb first-line treatment with the combination of Amivantamab plus Lazertinib in patients carrying classical EGFR mutations (exon 19 deletions or L858R on exon 21) [82]. Also, in this case, the combination improved the outcomes of the patients, suggesting that such kind of treatment could represent a new standard of care [83]. Finally, the MARIPOSA-2 trial (NCT04988295) studied the efficacy of Amivantamab in combination with chemotherapy with or without Lazertinib versus chemotherapy alone in patients with classical EGFR mutations who progressed on Osimertinib. This setting is particularly interesting for the application of Amivantamab, considering that a considerable fraction of patients developing Osimertinib resistance rely on MET amplification [84]. Results of the trial were encouraging, highlighting a clear improvement in progression-free survival [85]. Nevertheless, issues of toxicity requiring an accurate evaluation of the applicable drug dose regimen have been scored [86]. See Supplementary Table S2 for additional information about the mentioned trials with MET antibodies eliciting a response from the endogenous immune system.

## 4. Inhibition of MET Signaling to Modulate Cancer Immune Tolerance

It is well known that the endogenous immune response is subjected to positive and negative regulations. The discovery of immune checkpoint molecules deputed to maintain the complex regulation of T cell activity in physiological conditions represented a milestone in immunology. The consequent strategies developed to control immune checkpoint functions represented a breakthrough for therapeutic applications. The HGF/MET axis has been involved in regulating PD-L1/PD-L2 expression. MET amplification has been associated with high levels of PD-L1 expression in NSCLC [87,88], gastric carcinoma [89], malignant melanoma [90], and glioma [91] patients. Strong PD-L1 expression has also been found in tumors with MET exon 14 skipping [92] or MET overexpression [93,94]. These observational data suggest that cancers with MET alterations could potentially benefit from treatments targeting immune checkpoint molecules (Immune Checkpoint Inhibitors, ICIs). This was assessed in a retrospective study in which stage IV NSCLC MET exon 14 skipping patients with >50% PD-L1 expression treated as first-line therapy with Pembrolizumab showed an improved therapeutic outcome [95].

From a mechanistic point of view, MET activation triggers the MAP kinase pathway resulting in upregulation of the downstream NF-κBp65 transcription factor which, acting on the PD-L1 promoter, induces PD-L1 transcription [96]. This pathway is independent of JAK-2 kinase and is involved in both HGF-dependent and -independent MET signaling activation [97]. Notably, PD-L1 upregulation induced by MET amplification in EGFR-driven/erlotinib-treated NSCLC concurs to sustain secondary resistance to EGFR-TKI [98]. In cancer cells characterized by *MET* amplification, Interferon-gamma (INF-γ) treatment elicits intracellular signaling involving the JAK-Stat pathway that further induces the expression of PD-1 ligands [99]. In both cases, i.e., in the presence or the absence of INF-γ, MET-targeting treatment is sufficient to diminish PD-L1 levels, suggesting that cancer immune evasion can be limited by blocking MET signaling. Considering these results, the combination of MET-TKIs with ICIs should offer therapeutic advantages. Indeed, this has been proven pre-clinically in an orthotopic mouse model of pancreatic cancer, where the combination of Capmatinib and a PD-L1-targeting antibody showed therapeutic efficacy and a synergistic effect compared with both monotherapies [100]. Currently, the MET/ICI dual-targeting therapeutic strategy is under evaluation in clinical trials including NSCLC (NCT05782361; NCT04139317; NCT04323436; NCT03647488), oesogastric adenocarcinoma (NCT05135845), and melanoma (NCT03484923) patients. For some of these studies, MET alterations are an inclusion criterion. Ongoing results of these trials have outlined that a relevant number of patients experienced toxic effects, suggesting that a careful evaluation of the dose and the schedule of MET-TKIs and ICIs in combination is required. See Supplementary Table S3 for additional information about the mentioned MET/ICI-targeting trials.

**Figure 2:**
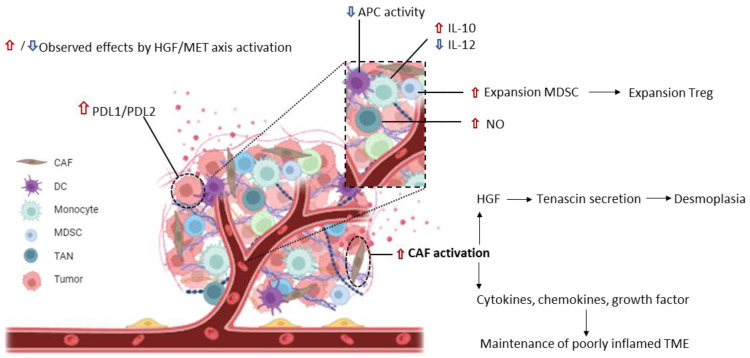
Schematic representation of the modulation exerted by the HGF/MET signaling on the immunosuppressive status of the tumor and its microenvironment. Red arrows represent an upregulation of immunosuppression induced upon HGF/MET axis activation; blue arrows indicate a reduction in immunostimulatory events caused by the axis. In the inset, a magnification shows immune cell populations infiltrating the tumor microenvironment. CAFs: Cancer-Associated Fibroblasts; DCs: Dendritic cells; MDSCs: Myeloid-Derived Suppressor Cells; TAN: Tumor-Associated Neutrophil; APCs: Antigen-Presenting Cells.

It is well known that in solid tumors cancer cells are embedded by the tumor stroma which favors their expansion, hampers the activity of exogenous drugs, and limits the immune response. The HGF/MET axis can modulate the immune suppressive tumor microenvironment (TME), acting at different levels. Activated Cancer-Associated Fibroblasts (CAFs) are a critical component of tumor stroma, producing much of the extracellular matrix. A robust fibrotic TME characterizes some tumors. Such dense fibrosis (desmoplasia) constitutes a physical obstacle to immune cell infiltration [101]. Activated CAFs secrete HGF, inducing MET activation in cancer cells, and these respond by increasing Tenascin (TNC) secretion. TNC boosts stroma activation, further exacerbating desmoplasia. The delivery of HGF/MET axis inhibitors can interrupt such a positive feedback loop, resulting in stroma rewiring [102]. Activated CAFs not only produce HGF but also secrete several cytokines, chemokines, and growth factors suitable to recruit and sequester various immune cells into the tumor, acting to maintain poorly inflamed/pro-tumoral environmental conditions. Thus, inhibition of the HGF/MET axis occurring on cancer cells and TME components will revert stroma activation. The TME includes cells of the immune compartment expressing MET such as Myeloid-Derived Suppressor Cells (MDSCs), monocytes, Neutrophils, and Dendritic cells (DCs). HGF treatment induces the expansion of MDSCs. As these cells inhibit the proliferation of anti-tumor lymphocytes and expand Treg, the HGF/MET axis significantly contributes to immunosuppression [103]. Treatment of monocytes with HGF stimulates the production of immunosuppressive IL-10 and reduces the secretion of immunostimulatory IL-12 [104]. Tumor-Associated Neutrophils (TANs) release Nitric Oxide (NO), which is detrimental to the activity of tumor-reactive T cells [105]. In conclusion, MET signaling favors tumor-tolerogenic conditions through direct and indirect modulations on cancer cells and the tumor microenvironment’s cellular components (Figure 2). Consequently, inhibition of MET signaling could benefit any therapeutic strategy involving immune system activation.

## 5. MET as a Target for Cellular Immunotherapy

Cellular immunotherapy is based on the treatment of patients with tumor-reactive immune effectors endowed with killing ability, previously expanded ex vivo. Often these immune cells are genetically manipulated to express either a recombinant T cell receptor or a CAR, a synthetic transmembrane protein built with an extracellular portion suitable to interact with a surface molecule expressed by the transformed cells and an intracellular portion deputed to the activation of the cytotoxic response. TCR and CAR expression enhance the immune effectors’ potency in hitting cancer cells. CAR-T immunotherapy achieved impressive success in the treatment of hematological malignancies [106]. Currently, a strong effort is put into reaching equally distinguished therapeutic results in solid tumors. One main point is to identify cancer-specific molecules in solid tumors to avoid the ‘on-target/off-tumor’ killing activity of the genetically modified immune effectors. Even if MET is not exclusively expressed by tumor cells, there is a difference in MET expression between normal and transformed cells. Indeed, adult normal tissues express any or low levels of MET that are increased only in the case of a response to critical conditions, such as tissue regeneration, while MET overexpression is a distinctive trait of a large portion of advanced malignancies. Another advantage of targeting MET by CAR immunotherapy is the potential hitting of the very inner roots of the tumor because MET expression is an inherent distinctive trait of cancer stem cells [17].

Formal proof of generating MET-CAR-T cells that can effectively kill cancer cells, sparing healthy tissue, has been provided in the work of Chiriaco et al. [107]. They showed that MET-CAR-T killing potency is dependent and proportional to the level of MET expression. Notably, a threshold of MET expression has been defined, under which CAR-T cells are not activated. Not all the MET-CARs could possess this feature, as precise characteristics of the synthetic receptor strongly influence the formation of a functional immune synapse. These features are: (i) CAR affinity for its specific target; (ii) the distance between the target and the effector cell when the interaction occurs, determined by the dimension of the entire extracellular domain composing the synthetic receptor. Some authors bypassed the evaluation of every MET-CAR construct specificity/selectivity by proposing MET-CAR-T delivery exclusively at the tumor site. For this reason, they selected cancers expressing high levels of MET treatable by locoregional CAR-T injection, i.e., malignant pleural mesothelioma and breast cancer, respectively [108,109]. Both studies showed MET-CAR-T therapeutic efficacy and safety in pre-clinical animal models. Although this kind of administration represents a prudent approach, it strongly limits the possible tumor types to which the therapy can be applied. Moreover, intra-tumoral administration does not guarantee the hit of distant lesions, which, on the contrary, are reachable in the case of systemic delivery. Interestingly, the strategy applied to breast cancer was based on a MET-CAR including the binding domain of Onartuzumab, and T cells have been engineered by mRNA electroporation. These features (i.e., using the sequence of a MET antibody already tested in the clinic and delivering T-cells that only transiently expressed the MET-CAR) considerably increased the intrinsic safety of the proposed therapeutic strategy, accelerating the transfer to humans. Indeed, RNA-CAR-T-cMET cells are under evaluation in the clinic for breast and melanoma patients (NCT03060356).

MET-CAR-T cells have been evaluated for hepatocellular, renal, and nasopharyngeal carcinomas [110,111,112]. The application to hepatocellular carcinomas has been refined by using a third-generation CAR, i.e., a construct that includes multiple co-stimulatory domains [113]. Although this type of CAR is more potent than the classical second-generation design, issues about their safety are still under debate because a stronger stimulation of T signaling can trigger a cytokine storm, an uncontrolled, highly detrimental systemic immune response [114]. In the case of papillary renal cell and nasopharyngeal cancers, MET-CAR-T cells have been tested alone and in combination with targeting molecules, the multi-targeting TKI Axitinib, and an EGFR inhibitor antibody Nimotuzumab. These combinations further enhance the therapeutic outcome. Although these studies did not clarify the precise mechanisms sustaining the treatment improvement, the authors did not refer to a possible direct effect exerted on the driver oncogene(s) by the targeting agents. Indeed, they considered that inhibition of VEGFR and EGFR was relevant to the function of the genetically modified T cells, favoring tumor infiltration, reducing the immunosuppressive status of the tumor microenvironment, and down-modulating PD-L1 expression in the cancer cells. Thus, these combinations could be effective in different types of tumors, as well as with CARs targeting molecules other than MET.

Gastric carcinoma is another malignancy suitable for MET-CAR-T treatment [107,115]. Gastric cancers are diseases in which *MET* amplification occurs with a relatively high frequency compared to other carcinomas [116]. Considering this feature, MET-targeting agents could be successfully applied to these patients, as proved by using antibodies or TKI in highly predictive preclinical models—such as patient-derived xenografts (PDX)—as well as in clinical cases [51,53,54,117,118,119,120]. Interestingly, Chiriaco et al. suggested the application of MET-CAR-T in gastric cancers not eligible for targeted therapy, due to a primary resistance, such as tumors with low *MET* amplification, or a secondary resistance, such as transformed cells developing activating mutations on downstream signal transducers under MET-TKI selective pressure [107].

**Figure 3:**
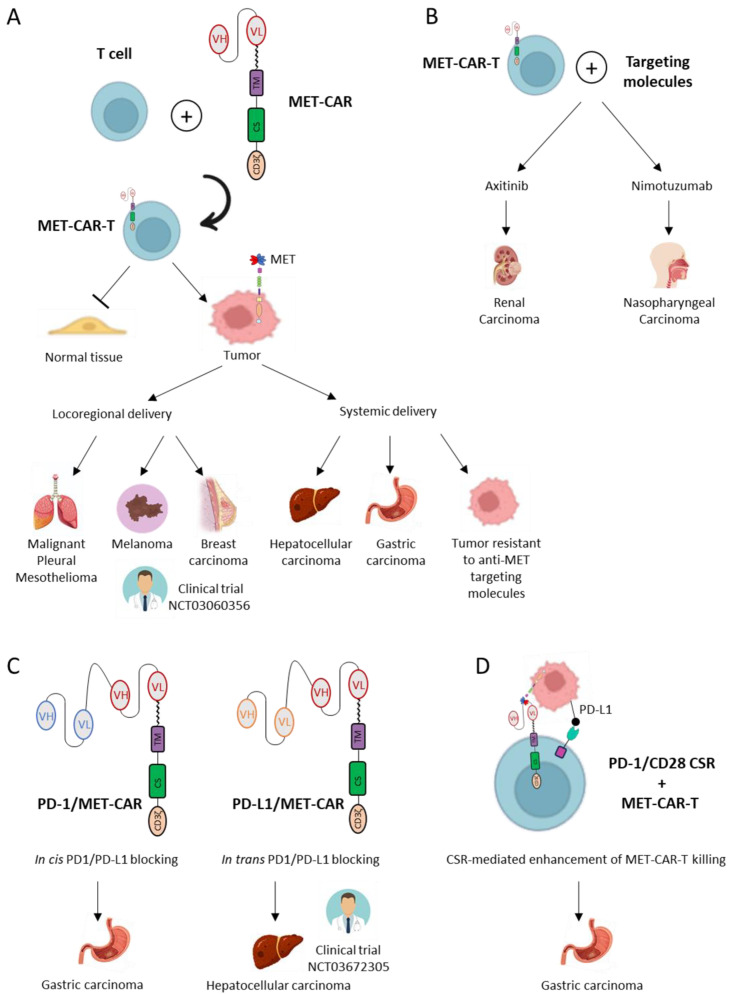
MET-CAR-T cellular immunotherapy designs and applications in different types of cancer. (**A**). MET-CAR-T cells are generated by genetic engineering of T lymphocytes with a CAR sequence. MET-CAR-T cells, sparing normal tissue while hitting MET-expressing tumor cells, have been tested in the indicated cancer models, either by locoregional delivery (**left**) or by systemic delivery (**right**). (**B**). MET-CAR-T cells have been applied in combination with targeting molecules (Axitinib or Nimotuzumab) in renal or nasopharyngeal carcinoma models, respectively. (**C**). Bispecific MET-CAR-T cells binding concomitantly with the MET receptor and an immune checkpoint molecule, PD-1 (**left**) or PD-L1 (**right**), have been evaluated against gastric or hepatocellular carcinoma, respectively. (**D**). T cells co-expressing a MET-CAR and a Chimeric Switch Receptor (CSR) have been investigated to potentiate the immune cell-mediated killing of gastric cancer cells. The therapeutic strategies evaluated in clinical trials are indicated in the figure.

MET-CAR-T immunotherapy faces the challenge of being applied to solid tumors. To improve the therapeutic outcome, some studies suggested delivering CAR-T cells capable of interfering with immune checkpoint molecules. Yuang et al. developed a dual-function MET/PD-1 CAR, including in the extracellular portion of the synthetic receptor an additional scFv region. This extracellular domain blocks the interaction of the MET-CAR-T cells with PD-L1 exposed on the surface of tumor cells through an in cis mechanism, by binding to PD-1 expressed by adjacent T-cells [121]. Similarly, another bispecific CAR has been designed by Jiang et al. In this case, the second scFv binds to PD-L1 on the surface of the target cells. This second strategy seems to be more efficacious because it not only blocks the PD-1/PD-L1 interaction but also triggers the cytotoxic response when the interaction with one or the other surface molecule occurs, limiting antigen escape effects [122]. The efficacy and safety of MET/PD-L1 CAR-T cells are under evaluation in the clinic for the treatment of hepatocellular carcinoma (NCT03672305). Finally, the co-expression of the MET-CAR with a PD1/CD28 chimeric switch receptor has been proposed. In this strategy, the inhibitory signal triggered by the PD-L1/PD-1 interaction is converted into an activation signal, because the switch receptor is composed of the extracellular region of PD-1 fused in frame with the transmembrane and intracellular domains of CD28. By expressing the PD-1/CD28 switch receptor, the authors showed that the anti-tumor efficacy of MET-CAR-T cells is potentiated, and the secretion of the inflammatory cytokine IL-6, one of the molecules responsible for the cytokine release syndrome, is reduced [123]. MET-CAR designs and applications are summarized in Figure 3. See Supplementary Table S4 for additional information about the mentioned MET-targeting cell therapy trials.

## 6. Conclusions

Since its discovery, the interest in the MET receptor has continuously grown and to date, it is considered a key oncogene in cancer. In the last twenty years, great effort has been made to develop molecules inhibiting MET. Initially, selective MET-TKIs were unavailable, increasing the risk of off-target toxicities, while now potent and specific small molecules have successfully entered the clinic. For what affects the antibodies, the simple inhibition of the ligand/receptor interaction did not result in positive therapeutic outcomes; thus, new antibodies endowed with multiple activities have been selected, tested, and preclinically validated. Even though clinical trials have been run or are currently running, outstanding therapeutic results with MET antibodies are still lacking. A step forward could be represented by the conversion of MET antibodies into ADCs, even if payload toxicity represents an issue to be solved. While MET exon 14 skipping patients represent the best target for selective MET-TKIs, MET antibodies still need to be coupled to the optimally responsive MET-altered cancer patients. Considering that MET could be activated by several genetic modifications, namely different forms of translocations, extracellular or intracellular point mutations, and variable levels of gene amplification, a clear-cut indication of when MET addiction is established is still lacking. This represents a crucial point to be solved as good therapeutic results can be reached only when patients responsive to the anti-MET therapy are correctly selected. Another point that still needs clarification is the role of HGF in the context of MET-mutated tumors. In principle, genetic aberrations sustain ligand-independent MET activation. However, HGF could further contribute to the aberrant MET signaling featuring the malignant phenotype. In this context, the possibility of a concomitant blocking of the ligand and the receptor could be a plus. MET overexpression is extremely common in cancer. This feature could represent an opportunity for largely applying MET-targeted therapies, considering the clear difference in MET expression between cancer and normal tissues. Molecules able to display a selective activity only in the presence of high surface MET levels could be used alone or in combination with other drugs. In this view, MET-ADCs and MET-CARs could represent precious tools to implement standard therapeutic options.

In the above-described scenario, exploiting the immune system for potentiating the therapeutic outcome is a highly interesting explorable route. MET plays an extremely complex role in cancer onset, cancer progression, and conditioning of the tumor microenvironment. Thus, advantages can emerge by exploring different immunotherapy approaches. MET addicted cancer therapy can benefit from antibodies exerting their inhibitory properties by activating antibody-dependent immune functions. MET-TKIs can be combined with treatments to reinforce the endogenous immune response by fighting cancer immune tolerance. MET-CAR cellular immunotherapy can represent a way to bypass primary and secondary resistances, offering a treatment opportunity for otherwise unmet clinical needs.

In conclusion, following the paradigm of precision medicine, the accurate evaluation of the HGF/MET axis function in every patient will allow the selection of the optimal MET-targeting immunotherapy approach to obtain the most powerful anti-tumor response.

## Figures and Tables

**Table 1:** Mechanisms of action of different MET antibodies.

Antibody	Structure	Epitope on MET	HGF Displacement	MET Downregulation	MET Shedding	Antibody-Dependent Immune Functions	Antibody Drug Conjugated	Clinical Trial *
Onartuzumab (OA-5D5, MetMab)	Monovalent IgG1/k	SEMA (blades 4/5/6)	Yes	No	No	No	No	Ph III
Telisotuzumab (ABT-700)	Bivalent IgG1/k	IPT-1	Yes + inhibition of receptor dimerization	Yes	Not reported	No	Teliso-V (ABBV-399) conjugated with Vedotin	Ph III (Telisotuzumab) Ph III (Teliso-V)
Emibetuzumab (LY 2875358)	Bivalent IgG4/k	SEMA (blades 2/3)	Yes	Yes	No	No	No	Ph II
Amivantamab (JNJ-61186372)	Bispecific (MET/EGFR) IgG1/k	SEMA (blade2)	Yes	Yes	Not reported	ADCC ADCP ADCT	No	Ph III
ARGX-111	Bivalent IgG1/k	SEMA (blades 2/3)	Yes	Yes	No	ADCC	No	Ph I
Sym015 (1:1 mix of Hu9006 and Hu9338)	Bivalent mAbs, IgG1/k	SEMA (blade 2, blade 3)	Yes	Yes	Not reported	ADCC CDC	No	Ph i/II
SAIT-301	Bivalent IgG2/k	SEMA (MET α-chain)	Yes	Yes	No	No		Ph I
REGN-5093 (METxMET)	Biparatopic IgG1,IgG3/k	SEMA (blade 3, blades 5/6)	Yes	Yes + inhibition of surface receptor recycling	Not reported	No	REGN-5093-M114 (METxMET-M114) conjugated with a maytansin derivative	Ph I/II (REGN-5093) Ph I/II (REGN-5093-M114)
hOA-DN30	Monovalent IgG1/k	IPT-4	No	No	Yes	No	No	IND completed; clinical trial expected in 2024 ^#^

* for each antibody, the most advanced clinical phase is reported, as described in May 2024 in www.clinicaltrials.gov (accessed on 15 May 2024). ^#^ the clinical trial will test a version of hOA-DN30 with some amino acid changes. ADCC: Antibody-Dependent Cellular Cytotoxicity; ADCP: Antibody-Dependent Cellular Phagocytosis; ADCT: Antibody-Dependent Cellular Trogocytosis; and CDC: Complement-Dependent Cytotoxicity.

## Supplementary Materials

The following supporting information can be downloaded at: https://www.mdpi.com/article/10.3390/ijms25116109/s1 [78,81,85,124,125,126,127,128,129].
