# Supplementary material for: MET Oncogene Targeting for Cancer Immunotherapy
Source: Int J Mol Sci. 2024 Jun 1;25(11):6109. doi: 10.3390/ijms25116109 (PMC11173045; doi:10.3390/ijms25116109)
Supplement: Supplementary file 1 [file ijms-25-06109-s001.zip › ijms-3015043-supplementary.pdf]

Supplementary Table S1. Clinical trials with MET-ADCs

Trial ID	Molecule	Combination	Comparator	Disease	Phase	Status*	Results
NCT05513703	Teliso-V	No	No	Advanced/Metastatic Non-Squamous NSCLC<br>MET gene amplification	Ph II	Active/Not recruiting	Not reported
NCT04928846	Teliso-V	No	Docetaxel	Previously Treated Non-Squamous NSCLC<br>MET overexpression	Ph III	Recruiting	Not reported
NCT02099058	Teliso-V	None<br>or<br>Erlotinib<br>or<br>Nivolumab<br>or<br>Osimertinb	No	Advanced NSCLC	Ph I	Active/Not recruiting	Safe and tolerated as monotherapy; antitumor activity in MET-positive patients [124,125]<br><br>Acceptable toxicity in combination with Erlotinib; encouraging antitumor activity in EGFR TKI-pretreated/EGFR-mutated/ MET-positive patients [126]<br><br>Tolerated in combination with Nivolumab; limited antitumor activity in MET-positive patients [127]
NCT06093503	Teliso-V	Osimertinib	Cisplatin+ Pemetrexed<br>or<br>Carboplatin+ Pemetrexed	Advanced/metastatic non-squamous NSCLC<br>MET overexpression	Ph III	Not yet recruiting	N/A
NCT04982224	REGN5093-M114	None<br>or<br>Cemiplimab	No	Advanced NSCLC<br>MET overexpression	Ph I/II	Recruiting	Not reported

* As described in May 2024 in www.clinicaltrials.gov;

ADCs: Antibody Drug Conjugates; NSCLC: Non Small Cell lung Cancer; N/A: Not Applicable

Supplementary Table S2. Clinical trials with MET-Antibodies

Trial ID	Molecule	Combination	Comparator	Disease	Phase	Status*	Results
NCT02648724	Sym-015	No	N/A	Advanced solid tumors	Ph I/II	Completed	DTLs: 0%<br>Basket cohort: 0% ORR<br>Met amplified and MET ex14 skipping NSCLC cohort: 25% ORR
NCT02055066	ARGX-111	No	N/A	Advanced solid tumors overexpressing MET	Ph I	Completed	Overall favorable safety and tolerability up to 3 mg/kg.<br>Total patient treated: 46% DCR<br>One gastric patient with MET amplification: PR (best responder) [128]
NCT04538664	Amivantamab	Pemetrexed +Carboplatin	Pemetrexed +Carboplatin	Advanced/ Metastatic NSCLC Exon20 ins EGFR	Ph III	Active/Not recruiting	Superior efficacy of the combination versus chemotherapy alone<br>(median PFS: 11.4 vs 6.7 months; ORR: 73% vs 47%) [81]
NCT04487080	Amivantamab	Lazertinib	Osimertinib	Advanced/ Metastatic NSCLC Exon19 del or Exon 21 L858R EGFR	Ph III	Active/Not recruiting	Higher toxicity of the combination vs monotherapy<br>(≥ Grade 3 AEs: 75% vs 43%);<br>Superior efficacy of the combination versus monotherapy<br>(median PFS: 23.7 vs 16.6 months) [129]
NCT04988295	Amivantamab	Lazertinib+ Pemetrexed +Carboplatin or Pemetrexed +Carboplatin	Pemetrexed +Carboplatin	Advanced/ Metastatic Non-squamous NSCLC Exon19 del or Exon 21 L858R EGFR; progressed on/after Osimertinib	Ph III	Active/Not recruiting	Higher toxicity of the Ami+Laze+chemo vs Ami+chemo vs chemo<br>(≥ Grade 3 AEs: 92% vs 72% vs 48%);<br>Superior efficacy of the Ami+Laze+chemo or Ami+chemo versus chemo<br>(median PFS: 8.3 vs 6.3 vs 4.2 months) [85]

* As described in May 2024 in www.clinicaltrials.gov

EGFR: Epidermal Growth Factor Receptor; DTLs: Dose-limiting Toxicities; ORR: Objective Response Rate; DCR: Disease Control Rate; PR: Partial Response; PFS: Progression Free Survival; AEs: Adverse Events

Supplementary Table S3. Clinical trials with MET-TKI in combination with ICI

Trial ID	Molecule	Combination	Comparator	Disease	Phase	Status*	Results
NCT05782361	Tepotinib	Pembrolizumab	N/A	Advanced cancer/ NSCLC MET ex14 skipping positive	Ph I	Recruiting	Not reported
NCT04139317	Capmatinib	Pembrolizumab	Pembrolizumab	Advanced/metastatic NSCLC PD-L1≥ 50%	Ph II	Terminated	Lack of tolerability of the combination
NCT04323436	Capmatinib	Spartalizumab	Capmatinib	Advanced/metastatic NSCLC MET ex14 skipping positive	Ph II	Terminated	Lack of tolerability of the combination
NCT03647488	Capmatinib	Spartalizumab	Docetaxel	Advanced/metastatic NSCLC	Ph II	Completed	The study was not opened in the randomized part<br>67% disease/clinical progression<br>27% Adverse events
NCT05135845	Capmatinib	Spartalizumab	N/A	Gastric/oesophageal adenocarcinoma Two arms: MET amplified (≥ 6 MET gene copies) or not	PhII	Suspended	Unfavorable toxicity profile
NCT03484923	Capmatinib	Spartalizumab	Spartalizumab + Ieramilimab or Cankinumab or Ribociclib	Advanced/metastatic Melanoma	PhII	Completed	The arm was not opened in the extension part.<br>65% disease progression<br>12% Adverse events

* As described in May 2024 in www.clinicaltrials.gov

TKI: tyrosine Kinase Inhibitor; ICI: Immune Checkpoint Inhibitor; NSCLC: Non Small Cell Lung Cancer;

Supplementary Table S4. Clinical trials with MET-CART

Trial ID	Molecule	Combination	Comparator	Disease	Phase	Status*	Results
NCT03060356	MET-CART	No	No	Stage III/IV melanoma or advanced/metastatic ER/PR negative, HER2 not-amplified breast carcinoma MET expression ≥ 30%	Ph I	Terminated	Safe and feasible<br>Best response: SD (4/7 treated patients)[130]
NCT03672305	MET/PD-L1-CART	No	No	Hepatocellular carcinoma MET-positive	Ph I	Unknown	Not reported

* As described in May 2024 in www.clinicaltrials.gov

CART: Chimeric Antigen Receptor T cells; NSCLC: Non Small Cell Lung Cancer; ER: Estrogen Receptor; PR: Progesterone Receptor; SD: Stable Disease
